# Analysis of expression and prognosis of KLK7 in ovarian cancer

**DOI:** 10.1515/med-2020-0139

**Published:** 2020-09-30

**Authors:** Erhua Chen, Huifang Zhu, Yue Yang, Ling Wang, Jianhua Zhang, Yonghong Han, Xiang Liu

**Affiliations:** Department of Pharmacy and Medicine Pharmacy, Jiang Su College of nursing, Huaian, 223005, China

**Keywords:** ovarian cancer, KLK7, oncomine

## Abstract

**Background:**

Ovarian cancer is one of the common malignant tumors in female reproductive organs. Kallikrein-related peptidase (KLK) 7 is a secreted serine peptidase that is related to different cancer. To investigate the expression and significance of KLK7 in ovarian cancer.

**Materials and methods:**

The expression of KLK7 in human ovarian cancer was evaluated by Oncomine and Cancer Cell Line Encyclopedia database. Then the co-expression genes relevant to the *KLK7* gene were analyzed by the Pearson correlation test. Finally, the impact of KLK7 on clinical prognosis was investigated in distinct subtypes of ovarian cancer patients by UALCAN database and Kaplan–Meier plotter database.

**Results:**

It was found that the expression of KLK7 was higher in ovarian cancer compared with other types of cancer, such as gastric cancer and pancreatic cancer. The expression of KLK7 was found to be increased in four various ovarian cancer data sets compared with the healthy tissues. In addition, upregulation of KLK7 expression was associated with age and cancer stage. Moreover, survival analysis revealed that higher KLK7 expression was negatively associated with progression-free survival.

**Conclusion:**

Knowledge of the expression of KLK7 may be useful for better understanding the outcome in ovarian cancer patients.

## 1 Introduction

Ovarian cancer is the highest mortality of gynecologic malignancy, and it carries a lifetime risk of approximately 2% for women [1]. As ovarian cancer is usually detected in advanced stages, it has poor prognosis even with many treatment options until now [2]. Increasing evidence has suggested that those diagnosed with early stage ovarian cancer may achieve a cure with adjuvant treatment [3]. Therefore, exploring a promising novel biomarker to improve the survival rate of ovarian cancer patients is urgent.

To date, 15 kallikrein-related peptidases (KLKs) family genes are found in the human genome. All of them take part in apoptosis, digestive system enzyme activation and coagulation–fibrinolysis [4]. KLK7 is also known as human stratum corneum chymotryptic enzyme because of its early detection in human skin [5]. In breast cancer, KLK7 was significantly downregulated in the sera of breast cancer and benign breast disease patients, implying a role in the pathogenesis of infiltrating ductal carcinoma [6]. It has been reported that *KLK7* and *KLK10* are two of the most upregulated genes in papillary thyroid carcinoma, which are involved in cell adhesion and extracellular matrix remodeling [7]. The overexpression of KLK7 can increase the proliferation abilities and promote migration and invasive behavior in pancreatic cancer cells [8]. Wang et al. [9] identify the KLK4–7 genes exert key modulatory effects on some other cancer-related genes at the mRNA and proteins in ovarian cancer cells and tissues. KLK7 secreted in the ovarian cancer microenvironment could in turn accelerate cancer progression [10]. However, little is known about the expression and prognostic role of KLK7 in ovarian cancer.

In our study, Oncomine has been systematically employed to explore the association of KLK7 mRNA expression with ovarian cancer patients. Meanwhile, the correlation of gene and the clinical value of KLK7 were shown by UALCAN database. Survival analysis of KLK7 was achieved based on Kaplan–Meier plotter.

## 2 Materials and methods

### 2.1 Oncomine analysis

The Oncomine is a public cancer database (www.oncomine.org) for filtering and missing data in our needs. In this study, we set the screening criteria as follows: “Cancer Type: Ovarian cancer;” “Gene: KLK7;” “Data Type: mRNA,” “Different analysis: cancer vs. normal.” We defined the cutoff as 0.01 and 1.5 for *p* values and fold changes, respectively, to exam the mRNA expression status of KLK7 in different cancer types.

### 2.2 Cancer cell line encyclopedia (CCLE) database

The CCLE database provides public access to genomic data, analysis and visualization for cell lines. The KLK7’s expression in different cell lines is verified by CCLE database.

### 2.3 UALCAN database

UALCAN is an interactive web resource based on level 3 RNA-seq and clinical data of 31 cancer types. We analyzed the expression profiles of KLK7 in normal and ovarian serous cystadenocarcinoma samples based on clinicopathologic parameters, such as cancer stage, age, race and tumor grade.

### 2.4 The Kaplan–Meier plotter

Kaplan–Meier plotter database was used to evaluate the prognostic significance of KLK7 mRNA expression, which provided the data on survival and gene expression. All patient were divided into two groups, according to the median expression (high and low expression), to analyze the overall survival (OS) and progression-free survival (PFS) by calculating the hazard ratio (HR), 95% confidence intervals (95% CI) and log rank *p* value.

## 3 Results

### 3.1 The mRNA expression levels of KLK7 in human cancers

**Figure 1:**
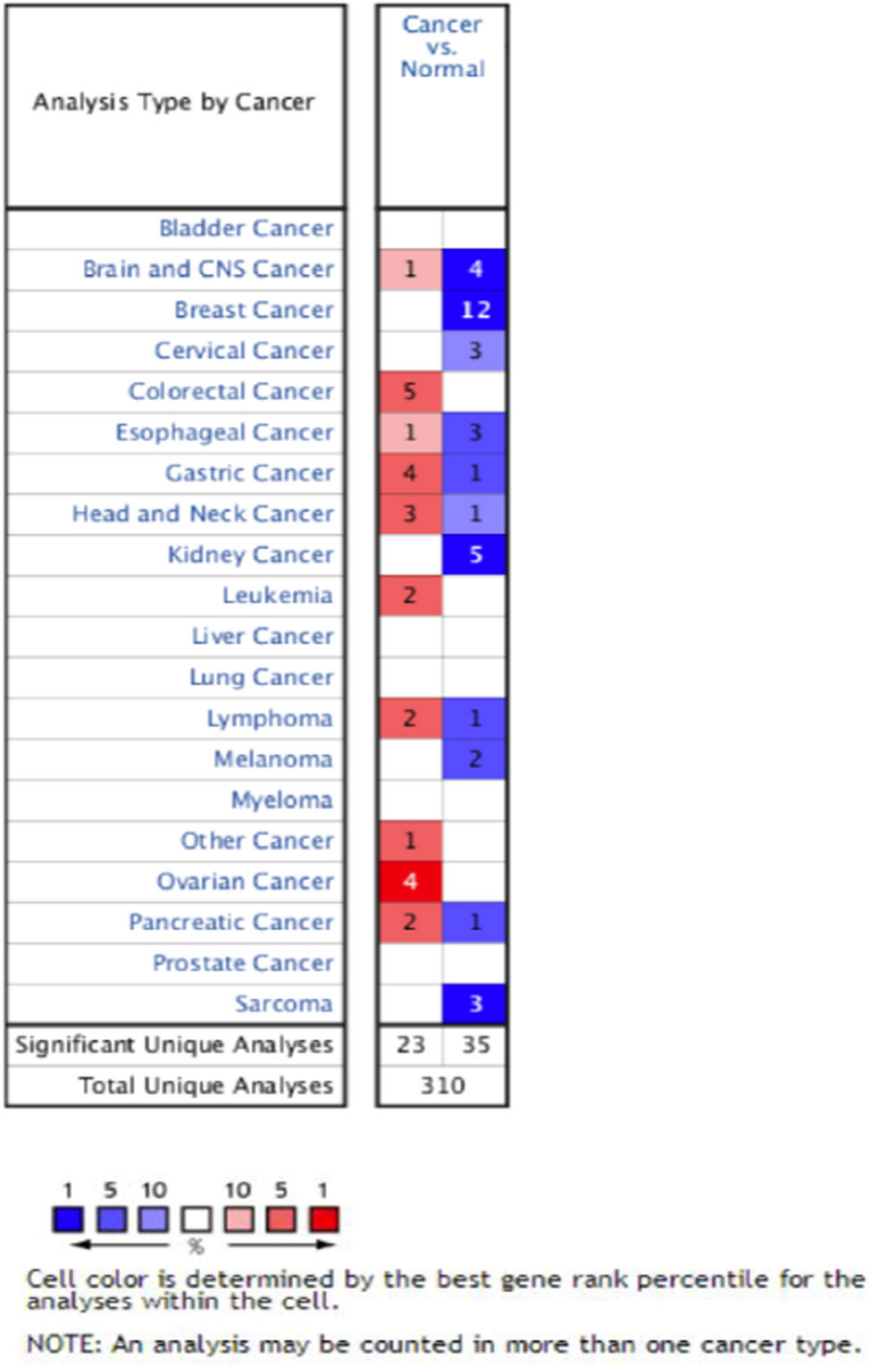
Transcriptional levels of KLK7 in different types of cancers (ONCOMINE).

To compare the mRNA expression difference of KLK7 between tumor and normal tissues in multiple cancers, we used the Oncomine database. As shown in Figure 1, a total of 310 analyses were included for KLK7. The KLK7 mRNA expression was upregulated in 23 studies and downregulated in 35 studies. The KLK7 expression showed upregulated in cancer tissues compared to that in normal tissues.

### 3.2 The expression level of KLK7 mRNA in human ovarian cancer

**Figure 2:**
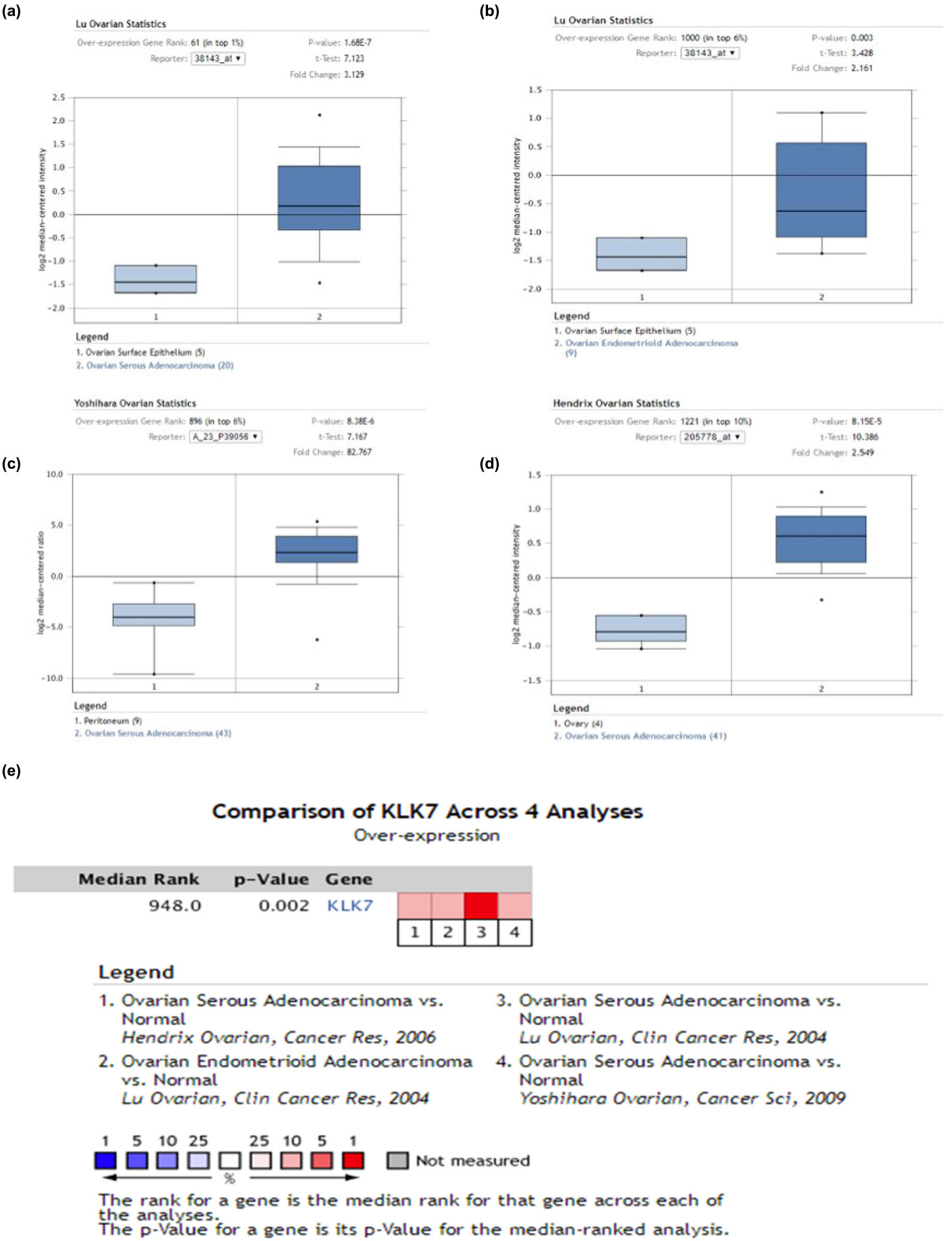
(a–d) Expression of KLK7 in in four various cancer data sets, (e) comparison of KLK7 across four analyses.

As shown in Figure 2a–d, KLK7 was found to be increased in four various cancer data sets compared with healthy tissues including ovarian serous adenocarcinoma [11] and ovarian endometrioid adenocarcinoma [12,13].

**Figure 3:**
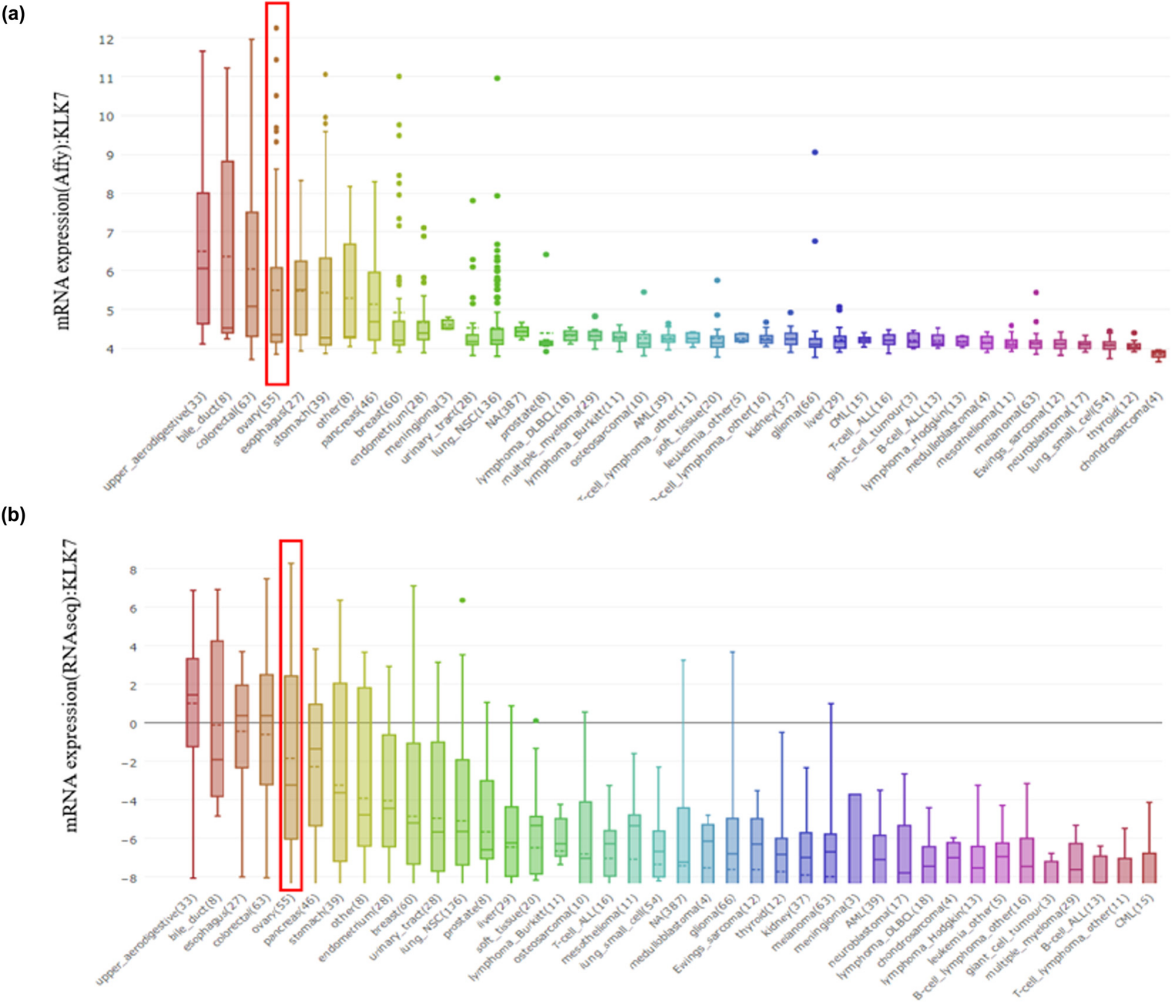
(a) Expression of KLK7 based on Affy gene chip data, (b) expression of KLK7 based on RNA-seq data.

A meta-analysis of the results of these four studies was studied. The KLK7 gene was ranked as 948.0 in all expressed genes, which means that KLK7 mRNA expression was significantly increased in two types of ovarian cancer (Figure 2e). Moreover, CCLE database analysis revealed that the mRNA expression levels of KLK7 in ovarian cancer compared with the other cancer cells. The mRNA expression of KLK7 ranks fourth highest in breast cancer and this is based on Affy gene chip data (Figure 3a). The mRNA expression of KLK7 ranks fifth highest in different tumor cell lines RNA-seq data, which is behind that of upper aerodigestive tract, bile duct, esophagus and colorectal (Figure 3b).

### 3.3 Expression correlation analysis of KLK7 in ovarian cancer

**Figure 4:**
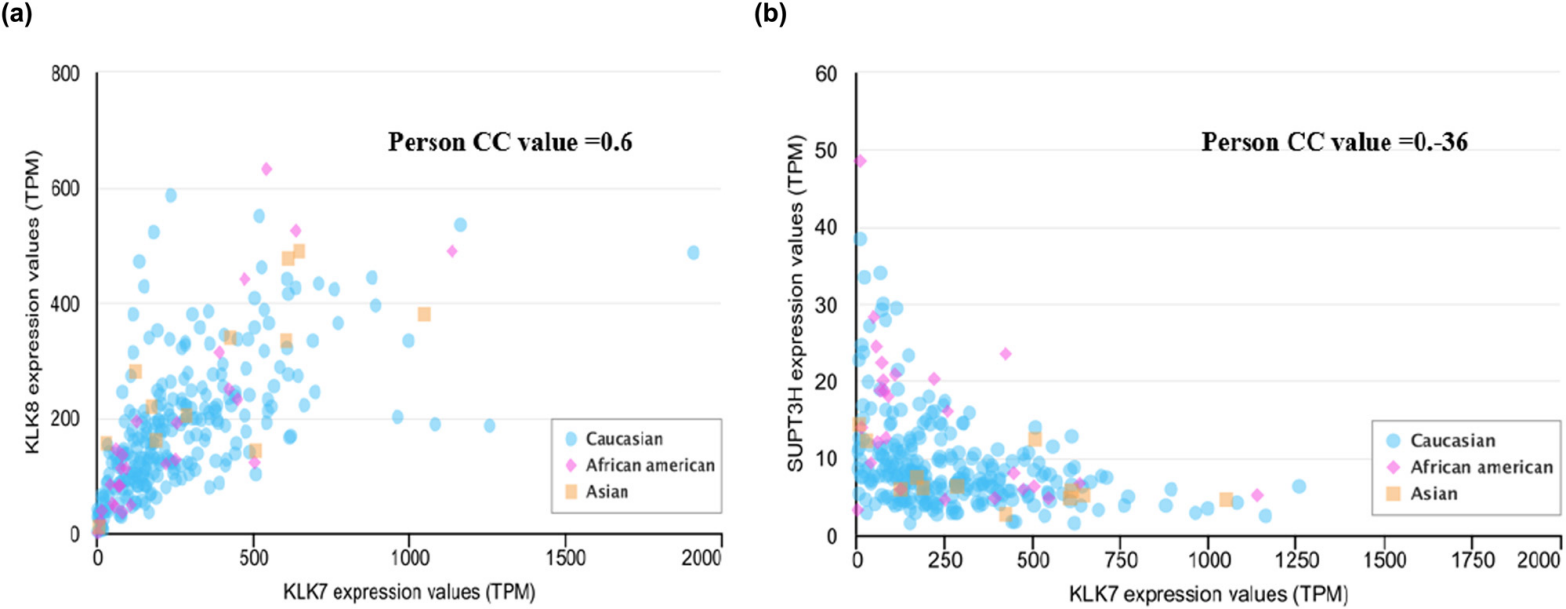
Correlation of *KLK8*, *SUPT3H* and *CHEK1* gene expression in ovarian cancer. (a) Gene expression correlation between KLK7 and KLK8 in OV, (b) gene expression correlation between KLK7 and SUPT3H in OV.

We selected top two genes (*KLK8* and *SUPT3H*) that most positively or negatively correlated with KLK7 to analysis. The *klk8* gene expression level was positively correlated with the KLK7 expression with a Pearson CC value of 0.6 (Figure 4a). We observed a negative correlation between KLK7 and SUPT3H expressions with a Pearson CC value of 0.36 (Figure 4b).

### 3.4 Association between *KLK7* gene expression and clinical pathological parameters in patients with ovarian cancer

**Figure 5:**
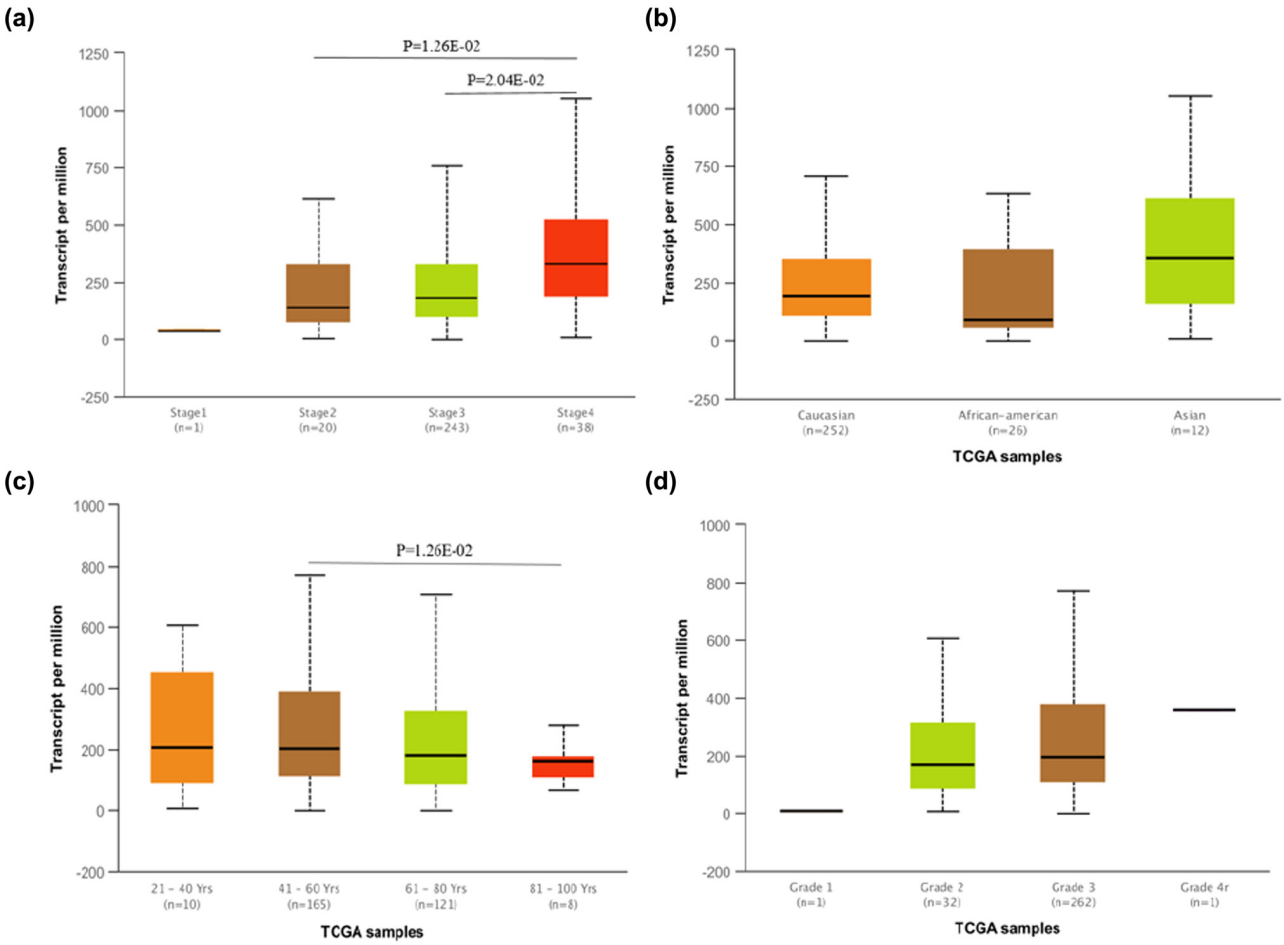
The relative expression of KLK7 in ovarian cancer. (a) Expression of KLK7 in OV based n individual cancer stages, (b) expression of KLK7 in OV based on patient's race, (c) expression of KLK7 in OV based on patient's age, (d) expression of KLK7 in OV based on tumor grade.

We next concentrated on the detection of KLK7 expression in normal and ovarian serous cystadenocarcinoma samples using UALCAN database. As shown in Figure 5, our analytical mining of the UALCAN database indicated that the expression level of KLK7 was higher in stage 4 compared with stages 2 and 3 of ovarian serous cystadenocarcinoma (*P* < 0.05) for cancer stages. Besides, KLK7 was downregulated in the age-group 81–100 years compared with those in the age-group 41–60 years (*P* < 0.05). Unfortunately, no significant difference was observed in the expression of the patients’ race and cancer grade.

### 3.5 KLK7 expression was correlated with the survival of ovarian cancer

**Figure 6:**
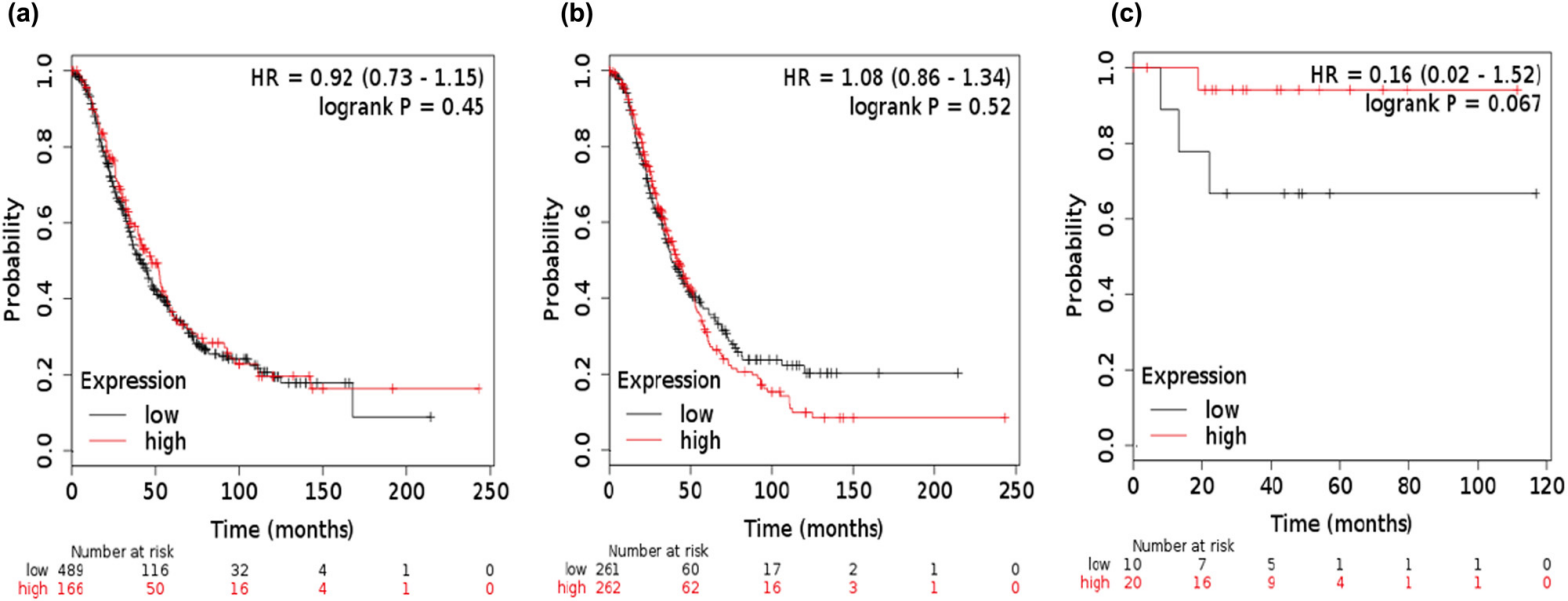
Prognostic value of KLK7 expression for OS of patients with ovarian cancer based on the Kaplan–Meier plotter. (a) All KLK7 (239381_at), (b) serous KLK7 (239381_at), (c) endometrioid KLK7 (239381_at).

**Figure 7:**
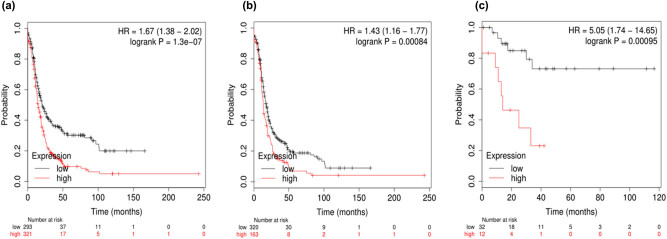
Prognostic value of KLK7 expression for PFS in patients with ovarian cancer based on the Kaplan–Meier plotter. (a) All KLK7 (239381_at), (b) serous KLK7 (239381_at), (c) endometriod KLK7 (239381_at).

To further assess the prognostic value of KLK7 in ovarian cancer, the KLK7 mRNA expression was evaluated; the Affymetrix ID was 239381_at KLK7. High KLK7 mRNA expression indicated unfavorable PFS for all patients with ovarian cancer (HR, 1.67; 95% CI, 1.38–2.02; *P* = 1.3 × 10^−07^; Figure 6a), patients with serous ovarian cancer (HR, 1.43; 95% CI, 1.16–1.77; *P* = 0.00084; Figure 6b) and patients with endometrioid ovarian cancer (HR, 5.05; 95% CI, 1.74–14.65; *P* = 0.00095; Figure 6c). KLK7 exhibited no association with OS in patients with serous ovarian cancer (Figure 7a), serous ovarian cancer (Figure 7b) and endometrioid ovarian cancer (Figure 7c). Although KLK7 expression was not significantly correlated with poor OS, the higher expression of KLK7 in all patients with ovarian cancers is significantly correlated with better PFS (*P* < 0.05).

## 4 Discussion

Most ovarian cancer patients cannot be cured completely, even with the combination of cytoreductive surgery and chemotherapy drugs [14]. Based on tissue analysis, KLK7 was found in both stromal and tumor cells, whose expression was higher in tumor cells [15,16]. Studies have found that KLK7 increases the expression of integrity adhesion receptors and both forms of the produced serine proteases (KLK7 and the nonproteolytic form) work in ovarian cancer peritoneal invasion [17]. Although some other studies have indicated that different members of KLK family associated with specific cancers and KLK7 as preferred targets for inhibition of ovarian cancer, the distinct role of KLK7 remains to be elucidated [18,19]. In our study, the expression and prognostic of KLK7 were systematically identified by several online databases.

Many studies have reported that KLK7 is overexpressed in healthy tissue than in tumors of ovarian cancer patients and have linked with the some other diseases [20,21] We first systematically analyzed the expression of KLK7 in different cancers using ONCOMINE, and our results confirmed that KLK7 was overexpressed in ovarian cancer, colorectal cancer and gastric cancer. Subsequently, our analysis suggested that high expression of KLK7 in ovarian cancer compared to normal controls among four data sets (Figure 2a–d). By the comparison of KLK7 expression in four data sets, meta-analysis demonstrated that KLK7 was significantly increased in two types of ovarian cancer, namely, ovarian serous adenocarcinoma and ovarian endometrioid adenocarcinoma (Figure 2e). Additionally, KLK7 was also highly expressed in human ovarian cancer cell lines, as implicated by CCLE database, supporting the critical role of KLK7 in ovarian cancer initiation or progression (Figure 3a and b).

Tamir et al. [22] suggested that KLK7 mRNA and protein overexpression are directly associated with early stage ovarian carcinomas and can be measured in patient tissue and serum samples. Further, Kyriakopoulou et al. [23] reported overexpressed KLK7 in ovarian cancer had an association with those high tumor grade diseases. In our report, we demonstrated that the expression of KLK7 was higher in a range of 41–60 years cancer stage. Previous studies indicated that high KLK7 expression was significantly associated with prolonged OS and PFS [24]. Then we evaluated whether overexpression of KLK7 was associated with clinicopathological features and survival outcomes by Kaplan–Meier plotter database. In contrast to this result, our finding revealed that overexpressed KLK7 was significantly associated with worse FPS in ovarian cancer.

Our study has certain limitations. It was only analyzed by online databases, and experimental or clinical validation would be needed to confirm the expression of KLK7 in ovarian cancer. More investigations should focus on a functional characterization and molecular mechanisms of the upregulated or downregulated factors in other significant KLK7 family members that are involved in KLK-mediated functions. In addition, we did not assess the potential diagnostic and therapeutic effect and explore whether it can be used as diagnostic markers or treatment. Taken together, we identified *KLK7* gene is potentially involved in prognosis through bioinformatics analysis. *KLK7* gene should be considered as potential prognostic biomarker for a better understanding of ovarian cancer progression and therapy.
